# Prevention of Juvenile Smoking

**Published:** 1893-01

**Authors:** 


### THE PREVENTION OF JUVENILE SMOKING.

It is time that the attention of all responsible persons should be seriously directed to the prevalence and increase of tobacco smoking among boys. Here and there, there have been observed expressions of a strong repugnance existing in the public mind against this form of juvenile perversity; but we still lack the support of a general and outspoken objection to its continuance. At the same time we feel assured that no man who has really given any thought to the matter would hesitate in condemning the injurious folly of this practice. Stunted growth, impaired digestion, palpitation, and the other evidences of nerve exhaustion and irritability have again and again impressed a lesson of abstinence which has hitherto been far too little regarded. A further stage of warning has been reached in a case which lately came before the coroner for Liverpool. A lad was in the habit of smoking cigarettes and cigar-ends, and, after an attack of sickness, died somewhat suddenly. The post-mortem examination revealed fatty changes in the heart, which there was little doubt, as the verdict held, had been fatally supplemented in their influence by the smoking habit referred to. This, of c ourse, is an extreme example. It is also, however, after all, only the strongly colored illustration of effects upon health which are daily realized in thousands of instances. We have no hesitation in asserting once more our conviction that it is incumbent upon the legislature, in view of its known pernicious effect upon mind and body during boyhood, to restrict this habit by an age limit which will fall outside this period.—*Lancet.*

